# Phospholipid Biosynthesis Genes and Susceptibility to Obesity: Analysis of Expression and Polymorphisms

**DOI:** 10.1371/journal.pone.0065303

**Published:** 2013-05-28

**Authors:** Neeraj K. Sharma, Kurt A. Langberg, Ashis K. Mondal, Swapan K. Das

**Affiliations:** Section on Endocrinology and Metabolism, Department of Internal Medicine, Wake Forest School of Medicine, Winston-Salem, North Carolina, United States of America; The Children's Hospital of Philadelphia, United States of America

## Abstract

Recent studies have identified links between phospholipid composition and altered cellular functions in animal models of obesity, but the involvement of phospholipid biosynthesis genes in human obesity are not well understood. We analyzed the transcript of four phospholipid biosynthesis genes in adipose and muscle from 170 subjects. We examined publicly available genome-wide association data from the GIANT and MAGIC cohorts to investigate the association of SNPs in these genes with obesity and glucose homeostasis traits, respectively. Trait-associated SNPs were genotyped to evaluate their roles in regulating expression in adipose. In adipose tissue, expression of *PEMT*, *PCYT1A*, and *PTDSS2* were positively correlated and *PCYT2* was negatively correlated with percent fat mass and body mass index (BMI). Among the polymorphisms in these genes, SNP rs4646404 in *PEMT* showed the strongest association (p = 3.07E-06) with waist-to-hip ratio (WHR) adjusted for BMI. The WHR-associated intronic SNP rs4646343 in the *PEMT* gene showed the strongest association with its expression in adipose. Allele “C” of this SNP was associated with higher WHR (p = 2.47E-05) and with higher expression (p = 4.10E-04). Our study shows that the expression of *PEMT* gene is high in obese insulin-resistant subjects. Intronic *cis*-regulatory polymorphisms may increase the genetic risk of obesity by modulating *PEMT* expression.

## Introduction

Endoplasmic reticulum (ER) is the major site of protein and lipid biosynthesis in cells [1]. A recent proteomic analysis revealed a shift in ER function from protein to lipid synthesis and metabolism in obese mice compared to lean animals. Further analysis revealed an increase in the phosphatidylcholine/phosphatidylethanolamine (PC/PE) ratio due to a shift in *de novo* lipogenesis in the obese ER lipidome [2]. This derangement in the composition of phospholipids was caused by transcriptional modulation of genes involved in phospholipid biosynthesis and remodeling [2]. An increased PC/PE ratio in the hepatic ER membrane perturbed ER calcium retention and homeostasis (by modulating sarco-endoplasmic reticulum calcium ATPAse –SERCA activity) leading to compromised protein folding and ER stress [2]. We and others have shown that activation of chronic ER stress in adipose and liver plays an important role in the development of obesity and related metabolic sequelae in human subjects [3]–[5]. However, the molecular and genetic regulatory mechanisms that lead to activation of chronic ER stress in obesity are not well understood.

Interestingly, mRNA and protein levels of phosphatidylethanolamine N-methyltransferase (*PEMT*), an enzyme that catalyzes conversion of PE to PC, are markedly increased (∼20 fold) in 3T3-L1 cells upon differentiation to adipocytes [6]. Thus, *PEMT* may play an important role in biogenesis of adipocyte lipid droplets and thereby modulate fat mass *in vivo*. Compared to wild-type mice, *PEMT*-deficient mice (Pemt ^-/-^) are resistant to diet-induced hypertrophy of adipocytes in white adipose tissue and are also protected against high fat diet-induced obesity and insulin resistance [7]. The ethanolamine-phosphate cytidylyltransferase 2 (*PCYT2*) gene is involved in synthesis of PE from diacylglycerol. Interestingly, mice deficient in *PCYT2* (*Pcyt2* ^+/−^) are obese and insulin resistant [8]. However, the role of these phospholipid biosynthesis and remodeling genes in human obesity is not well understood.

Given these considerations, we hypothesized that adipose and muscle tissue expression of genes in the ER phospholipid biosynthesis pathway will be associated with obesity and insulin resistance in humans, and *cis*- regulatory polymorphisms (eSNPs) may increase susceptibility to obesity and insulin resistance by modulating expression of these genes. To test our hypothesis, we analyzed expression of four major genes (*PCYT1A*, *PCYT2*, *PEMT*, and *PTDSS2*) involved in ER membrane phospholipid biosynthesis/remodeling in adipose and muscle from 170 individuals with a broad range of insulin sensitivity (S_I_) and body mass index (BMI) values. We investigated the association of SNPs in these genes with obesity and glycemic traits in publicly available data from large genome-wide association studies. Finally, we evaluated the role of obesity-associated SNPs in these genes in regulating expression in adipose tissue.

## Materials and Methods

### Study subjects

Gene expression and effects of genotype on gene expression were studied in adipose and muscle tissue from 170 Caucasian or African American subjects recruited by advertisement. Subjects were in good general health, between 19–60 years of age, and had a body mass index (BMI) between 19 and 45 kg/m^2^. Detailed characteristics of the subjects in this study are described elsewhere [9]. All study participants provided written, informed consent under protocols originally approved by University of Arkansas for Medical Sciences (UAMS). Our current study was approved by Wake Forest School of Medicine Institutional Review Board (Protocol IRB00011083). Characteristics of the cohort are summarized in **Table 1**.

**Table 1:** Characteristics of study population.

	N	Mean ± SD
Gender (M/F)	170 (74/96)	
N (T2D/Non-diabetic)	170 (13/157)	
Ethnicity (C/AA)	170 (123/47)	
Age (yrs)	170	41.0±10.4
WHR	167	0.90±0.15
BMI (kg/m^2^)	170	29.5±5.8
PFAT	169	32.3±9.8
S_I_ (×10^−4^.min^−1^ [µU/ml]^−1^) *	136	3.7±2.2

* , Metabolic traits from FSIVGT evaluation of non-diabetic individuals. S_I_, insulin sensitivity index, Units are taken from MINMOD program. C, Caucasian; AA, African American; WHR, waist-to-hip ratio; BMI, body mass index; PFAT, percent of fat mass (measured by DEXA).

### Clinical Methods

Genomic DNA was isolated from blood samples using a Puregene DNA extraction kit (Qiagen Inc., Valencia, CA). Needle biopsies of abdominal subcutaneous adipose tissue and *vastus lateralis* muscle of all subjects were obtained under fasting conditions, and insulin sensitivity of non-diabetic subjects were evaluated by an insulin modified frequently sampled intravenous glucose tolerance test (FSIVGT). Body fat was determined by dual X-ray absorptiometry (DXA) scans (Hologic QDR-4500). Biopsy samples were quick frozen in liquid nitrogen and stored at −80°C for further use. The adipocyte fraction (AF) was separated from the stromal-vascular fraction (SVF) after collagenase digestion of freshly collected adipose biopsy samples, as described earlier [5]. Fresh tissue for AF and SVF separation was available only for a subset of 40 randomly selected non-diabetic subjects.

### Laboratory measurements

Insulin was measured by the UAMS Clinical Research Center core laboratory using an immuno-chemiluminometric assay (Invitron Limited, Monmouth, UK formerly Molecular Light Technology, Wales, UK). Assays with <10% CV were used for the analysis. Plasma glucose was measured by using a glucose oxidase method at LabCorp, Inc. (Burlington, NC).

### RNA extraction

Total RNA was isolated from whole adipose and adipocyte fractions using the RNAeasy Lipid Tissue Mini kit (Qiagen). Total RNA from the SVF of adipose was isolated using an RNAqueous kit (Ambion, Inc., Austin, TX). RNA from muscle biopsies was isolated using an Ultrasoec RNA kit (Biotecx Laboratories, Inc., Houston, TX). The quantity and quality of the isolated total RNA samples were determined by ultraviolet spectrophotometry (Nanodrop, Thermo Scientific, Pittsburgh, PA) and electrophoresis (Experion nucleic acid analyzer, BioRad Laboratories, Inc., Hercules, CA), respectively.

### Gene expression

A recent study by Fu et al in animal models identified an increase in the cellular PC/PE ratio as a mechanism proximal to the ER stress induction in obesity [2]. Transcription modulation of four genes involved in phospholipid biosynthesis and remodeling was the key factor for this increase in PC/PE ratio (**Figure S1**). Choline-phosphate cytidylyltransferase A (*PCYT1A*) and ethanolamine-phosphate cytidylyltransferase 2 (*PCYT2*) genes are involved in synthesis of PC and PE, respectively, from diacylglycerol. Phosphatidylethanolamine N-methyltransferase (*PEMT*) catalyzes the conversion of PE to PC by a sequential methylation reaction [10], while phosphatidylserine synthase 2 (*PTDSS2*) converts PE to phosphatidylserine (PS). Thus, we analyzed expression of these genes in tissues relevant to obesity and related metabolic phenotypes.

Total RNA (1 µg) was reverse transcribed using a Qiagen QuantiTect reverse transcription kit (Qiagen) following the manufacturer's protocol, which includes a DNase digestion step to remove genomic DNA contamination. Transcripts were measured by quantitative real-time PCR using Power SYBR green chemistry (Applied Biosystems, Inc., Foster City, CA) and normalized either to the expression of human ribosomal protein, large P0 (RPLP0 or 36B4) gene (for adipose and AF), or to 18S ribosomal RNA (for muscle and SVF), similar to our published studies [11]. The standard curves were generated for absolute quantification using pooled cDNA from the samples assayed. Transcript-specific oligonucleotide primers for real-time PCR were designed to capture most known splice variants and amplicons spanned an intron (see **table S1** for details). We have successfully generated expression data in adipose tissue of 155 subjects and in muscle of 160 subjects from our cohort.

### Association of single nucleotide polymorphisms (SNPs) in phospholipid remodeling genes with obesity and glucose homeostasis traits

To investigate whether the SNPs in genes in phospholipid remodeling were associated with obesity and glucose homeostasis traits, we examined publicly accessible data from large genome-wide association (GWA) studies in Caucasian subjects. We used meta-analyses of association data for BMI and WHR adjusted for BMI from the GIANT (http://www.broadinstitute.org/collaboration/giant/) consortium [12], [13]. In GIANT, summary statistics (including p-values and direction of effect for alleles) for BMI were available from a meta-analysis of up to 123,866 nondiabetic Caucasian participants for the genes of interest, while summary statistics for WHR adjusted for BMI phenotype were available for up to 77,169 nondiabetic Caucasian individuals. Data for glucose homeostasis traits were taken from the MAGIC dataset (http://www.magicinvestigators.org) [14], [15]. In MAGIC, fasting glucose and insulin resistance [homeostatic model assessment-insulin resistance (HOMA-IR)] data sets were generated by performing a meta-analysis of up to 21 genome-wide association studies in up to 46,186 nondiabetic Caucasian participants (summary statistics including effect allele, effect size and p-values were available). Two-hour glucose data sets were generated by a meta-analysis of nine genome-wide association studies in 15,234 nondiabetic Caucasian individuals.

We first examined the GIANT and MAGIC data for associations of SNPs within ±2 kb of four genes that modulate phospholipids in subcutaneous adipose tissue. The GIANT and MAGIC datasets included summary statistics for 115–119 SNPs for these regions. Data for 115 SNPs were available for WHR and 2hr glucose traits; for other traits, data for 119 SNPs were available. We considered a p-value of ≤0.0004 as significant (considering multiple testing corrections for 119 SNPs in our study). We further expanded our search to ±500 kb to identify any associations within the putative *cis*-regulatory region of these genes.

### SNP selection and genotyping in expression cohort

Resent studies have shown that complex trait-associated SNPs are enriched for regulatory SNPs (eSNPs) [16]. Thus, to gain more mechanistic insight, our next goal was to find out if trait-associated SNPs within these genes increases genetic predisposition to metabolic traits by modulating their expression. We found no significant associations of *PCYT1A*, *PCYT2*, or *PTDSS2* SNPs with metabolic phenotypes in the GIANT and MAGIC data sets (at the p≤0.0004 level). However, nine SNPs in *PEMT* showed significant associations (see Results). Thus, genotype vs. expression association was tested only for these SNPs in *PEMT*. Linkage disequilibrium between SNPs was evaluated by HaploView software [17] using genotyping data of CEU subjects from the HapMap project (http://hapmap.ncbi.nlm.nih.gov/). Three SNPs were selected to tag (r^2^>0.8) nine SNPs within ±2 Kb of the *PEMT* gene that were associated with WHR (adjusted for BMI). A coding non-synonymous SNP (rs7946) that showed a marginal association with WHR was also genotyped.

Genomic DNA isolated from total blood samples of individuals from our expression cohort was used for genotyping. Four SNPs were genotyped by Pyrosequencing (PSQ96, Qiagen Inc.; formerly Biotage, Sweden) using a modification of the manufacturer's protocol including a biotinylated universal primer. Details of genotyping assays are given in **table S2**.

### Statistical analysis

We used the MINMOD Millennium program to analyze FSIVGT data to determine insulin sensitivity (S_I_) of non-diabetic subjects [18]. Gene expression data and all skewed variables were normalized through logarithm transformation and used in all analyses. Partial correlation measures of obesity traits (BMI, WHR, and percent of fat mass) and insulin sensitivity (S_I_) with gene expression were calculated after controlling for age, gender, and self-identified ethnicity [5]. Since BMI and S_I_ are strongly correlated, additional adjustments for BMI were also included to evaluate correlations between gene expression and S_I_. Pre-computed p-values from the GIANT and MAGIC meta-analyses were used and queried using a Microsoft Access database to evaluate the association between SNPs in the four phospholipid remodeling genes and obesity and glucose homeostasis traits, respectively. Genotypic effects on gene expression were analyzed by one-way analysis of variance (ANOVA). Associations between genotype and gene expression were further evaluated by univariate analysis of variance using a general linear model (GLM) procedure, which includes age as a covariate and genotype, gender, and self-identified ethnicity as fixed factors [19]. All statistical analyses were implemented in SPSS v.13.0 (SPSS Inc., Chicago, IL). For gene expression analysis, we considered p<0.05 to be significant without correcting for multiple statistical tests, based on our strong *a priori* hypothesis and the high correlation among tested traits.

## Results

### Expression of phospholipid remodeling genes in adipose tissue is correlated with adiposity

Expression of *PCYT1A* (r = 0.36, p = 1.09×10^−5^), *PEMT* (r = 0.46, p = 9.57×10^−9^) and *PTDSS2* (r = 0.29, p = 4.72×10^−4^) in adipose showed significant positive correlations, while *PCYT2* (r = −0.30, p = 4.15×10^−4^) was negatively correlated with percent of fat mass (PFAT) and BMI in 142 non-diabetic subjects (**Table 2**). These results remained consistent even after using our entire sample, which included both diabetic and non-diabetic subjects (N = 155, data not shown). Further examination in adipose tissue fractions from a subset of 40 non-diabetic subjects indicated that the correlation of *PEMT* with adiposity was conferred by adipocytes (r = 0.38, p = 0.02) but not by cells in the stromal vascular fraction (Table 2). We also noted that the average expression of *PEMT* in adipocyte fractions was higher (∼2.5 Ct, t-test p = 1.15×10^−9^) than in stromal vascular fractions of adipose tissue.

**Table 2:** Correlation of metabolic traits with expression of phospholipid biosynthesis genes in different tissues of non-diabetic subjects.

Tissue	Gene		BMI	WHR	PFAT	S_I_	S_I_*
**Adipose**		**N**	142	138	142	131	131
	*PCYT2*	**r**	−0.34	−0.18	−0.30	0.34	0.25
		**p**	3.60E-05	0.039	4.15E-04	7.15E-05	0.005
	*PCYT1A*	**r**	0.26	0.24	0.36	−0.05	0.08
		**p**	0.002	0.004	1.09E-05	0.571	0.383
	*PEMT*	**r**	0.43	0.37	**0.46**	−0.33	−0.19
		**p**	1.12E-07	8.83E-06	**9.57E-09**	1.37E-04	0.029
	*PTDSS2*	**r**	0.21	0.24	0.29	0.04	0.15
		**p**	0.014	0.006	4.72E-04	0.675	0.094
**Muscle**		**N**	147	143	147	127	127
	*PCYT2*	**r**	−0.12	−0.11	−0.12	0.28	0.26
		**p**	0.16	0.18	0.15	0.002	0.005
	*PCYT1A*	**r**	−0.03	0.04	0.04	0.15	0.14
		**p**	0.75	0.63	0.67	0.10	0.12
	*PEMT*	**r**	−0.01	−0.04	−0.02	0.16	0.17
		**p**	0.92	0.67	0.80	0.07	0.06
	*PTDSS2*	**r**	0.01	0.00	0.01	0.12	0.13
		**p**	0.93	0.997	0.88	0.18	0.14
**SVF**		**N**	40	39	40	37	37
	*PEMT*	**r**	−0.13	0.26	0.05	−0.03	−0.11
		**p**	0.44	0.13	0.76	0.88	0.54
**Adipocyte**		**N**	39	38	39	36	36
	*PEMT*	**r**	0.37	0.57	0.38	−0.34	−0.19
		**p**	0.024	3.68E-04	0.022	0.051	0.30

r, partial correlation after controlling for age, gender, ethnicity; *, partial correlation after controlling for age, gender, ethnicity, and ln(BMI); BMI, body mass index; WHR, waist-to-hip ratio; PFAT, percent of fat mass (measured by DXA). N =  sample number, p =  p-value; SVF, stromal vascular fraction.

The FSIVGT-derived index of whole body insulin sensitivity (S_I_) was negatively correlated with expression of PEMT (r = −0.33, p = 1.37×10^−4^) and positively correlated with *PCYT2* (r = 0.34, p = 7.15×10^−5^) in adipose. However, correlations of *PEMT* and *PCYT2* expression with S_I_ were only marginally significant after adjustment for BMI (Table 2). *PCYT1A* and *PTDSS2* expression in adipose showed no significant correlations with S_I_.

### Phospholipid remodeling genes in skeletal muscle tissue

Average expression of *PEMT* was lower (∼3.5Ct, t-test p = 4.06×10^−83^) in skeletal muscle compared to subcutaneous adipose, and the other three genes tested in muscle showed similar levels of expression compared to adipose. Expression of these genes in muscle showed no significant correlation with expression in adipose tissue from the same subjects. Expression of these phospholipid remodeling genes in muscle was not correlated with obesity measures (Table 2). However, *PCYT2* expression in muscle was marginally correlated (r = 0.26, p = 0.005) with S_I_.

### PEMT gene polymorphisms are associated with adiposity

Interrogation of the meta-analysis data from the GIANT consortium identified 115 SNPs within ±2 kb of the *PTDSS2*, *PEMT*, and *PCYT1A* genes. None of these SNPs showed significant association at a genome-wide significance threshold (p<5.0×10^−8^). However, at our overall significance threshold (p ≤0.0004), 9 SNPs in *PEMT* showed significant associations with WHR adjusted for BMI (**Figure S2**). Further expansion of this search identified a locus in chromosome 17 significantly associated with WHR (adjusted for BMI). SNP rs4646404, located in an intron of *PEMT*, showed the strongest association (p = 3.07×10^−6^, “G” allele associated with increased WHR in 77,158 Caucasian subjects) within ±500 Kb of the gene (**Figure S3**). None of the SNPs within ±2 Kb of these genes showed any evidence for association with BMI in the GIANT meta-analysis dataset at the p≤0.00004 threshold. A similar search in meta-analysis data from the MAGIC consortium also identified no SNPs significantly associated with glucose homeostasis traits (including fasting plasma glucose and HOMA-IR in meta-analysis of 46,186 non-diabetic Caucasian subjects and 2 hr glucose tolerance in 15,234 subjects). No SNP association data were available for the ±2 kb region around the *PCYT2* gene. However, no SNP reached significance in an expanded search within ±500 kb region of this gene.

### Adiposity-associated variants in the PEMT gene are *cis*-eSNPs

**Figure 1:**
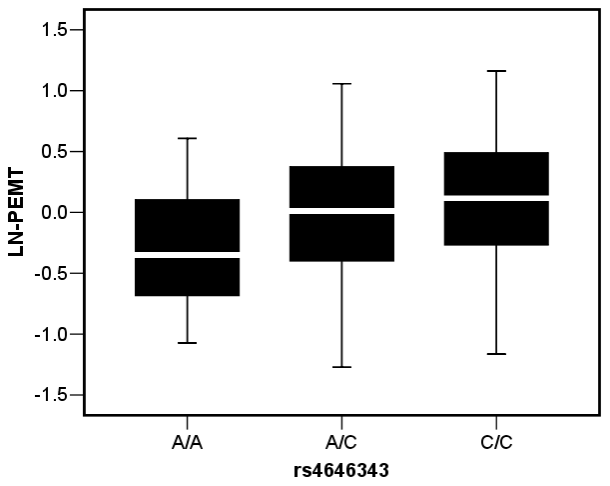
Genotypic association of SNP rs4646343 with PEMT expression in subcutaneous adipose. The box plot shows transcript level expression of PEMT for different genotypes (AA, n = 26; AC, n = 59 and CC, n = 69). PEMT expression is shown after 36B4 normalization and ln transformation. The box represents the interquartile range, which contains 50% of the values. The whiskers are lines that extend the box to the highest and lowest values, excluding outliers. A line across the box indicates the median.

We further investigated if the WHR-associated SNPs (within ±2 kb of PEMT gene) are *cis*-regulators in adipose tissue. In our cohort (N = 154), intronic SNP rs4646343 showed the strongest genotypic association (p = 9.16×10^−4^) with expression of PEMT transcript (**Table 3** **and** **Figure 1**). This association also remained significant (p = 4.10×10^−4^) in a non-diabetic Caucasian subset (N = 101) of our cohort. The SNP rs4646404 that showed the strongest association with WHR in the GIANT dataset was marginally associated (p = 0.024–0.09) with *PEMT* expression in adipose. We noticed that for all of these SNPs, alleles linked to increased WHR were associated with increased expression of PEMT.

**Table 3:** WHR-associated SNPs in *PEMT* modulate its transcript level expression in subcutaneous adipose tissue.

					All (N = 154)				Caucasian non diabetics (N = 101)		
SNP	Location (hg19)	Annotation	Allele1*	WHR* P-value	Genotype count	Allele1*	P	Padj	Genotype count	P	Padj
s7946	7409560	missense	G	0.016	AA/AG/GG	G	0.151	0.16	AA/AG/GG	0.023	0.080
		V 175 M			64/74/16				53/45/3		
s4646404	17420199	intron	G	3.07E-06	GG/AG/AA	G	0.053	0.09	GG/AG/AA	0.024	0.068
					97/44/12				54/35/11		
s897453	17425631	missense	C	6.00E-05	CC/TC/TT	C	0.016	0.008	CC/TC/TT	0.010	0.009
		V 58 I			77/55/21				39/42/20		
s4646343	17492077	intron	C	2.47E-05	CC/AC/AA	C	0.004	9.16E-04	CC/AC/AA	6.21E-04	4.10E-04
					69/59/26				37/42/22		

* , Allele 1, Trait increasing allele; P, ANOVA P-value for transcript expression level; P adj, statistical significance of genotypic association from univariate analysis of variance using a general linear model (GLM) procedure that included age as covariates and genotype, gender, ethnicity as fixed factors; WHR P-value, P-value for WHR adjusted for BMI as in GIANT data.

## Discussion

Studies by our laboratory and others have shown the induction of chronic ER stress in adipose tissue of obese subjects [3]–[5]. In concordance with recent animal studies [2], our study in human subjects now shows the transcriptional upregulation of genes involved in PC biosynthesis (including *PEMT*) and downregulation of the *PCYT2* gene, involved in PE biosynthesis, in the adipose tissue of obese subjects. Although we have not measured tissue phospholipid levels, the transcriptional profile of four genes indicates a possible increase in PC/PE ratio in adipose and may be a mechanism behind the perturbation of ER homeostasis and induction of ER stress. In our study, induction of *PEMT* in obesity was limited to adipocytes, and was not seen in muscle or cells in the SVF of adipose. Thus, similar to rodent models [6], [7], [20], induction of PEMT may also be mechanistically involved in stabilization of lipid droplets and hypertrophy of adipocytes in obese human subjects.

We attempted to elucidate the function of four phospholipid biosynthesis genes in obesity and related traits by combining phenotype, gene expression in tissues important for obesity, and genotype data in a metabolically well-characterized cohort. Integration of publicly available genome-wide association (GWA) findings with our study significantly expedited our search to identify functional polymorphisms that increase susceptibility to obesity by modulating expression at the transcript level. However, a notable limitation of our study is that we used only adipose and muscle samples. As noted previously, phospholipid remodeling in hepatic ER was the focus of a rodent model study [2]. Expression of PC/PE remodeling genes, including PEMT, is higher in liver [20] than in adipose and may be more predictive of obesity.

Although dietary and lifestyle risk factors play important roles, the contribution of genetic susceptibility to obesity is well established. Recent GWA studies have identified several susceptibility loci for obesity [12], [13], but few loci have been linked to a molecular mechanism. The GIANT consortium identified 16 associated (p<1.4×10^−6^) loci using a fixed effect meta-analysis of WHR from 77,167 subjects from 32 studies, employing study-specific linear regression adjusted for BMI and age, stratified by gender, and using an additive genetic model [12]. No SNPs in cellular phospholipid metabolism loci were reported by that study. The multiple testing corrections utilized in genome-wide statistical analysis allows detection of only the strongest effects and penalize weaker associations that may be biologically meaningful [21], [22]. In a focused search for genetic association of polymorphisms in phospholipid modulating genes, we identified an association of the *PEMT* polymorphism with WHR adjusted for BMI in the GIANT consortium dataset. The strongest association p-value (p = 3.07×0^−6^) for WHR within ±500 Kb of the *PEMT* gene is below the threshold and thus was not reported by GIANT consortium. Prioritizing genes that are causally involved with susceptibility to obesity may thus be helpful in identifying additional genetic susceptibility loci from GWA studies.

BMI is the most commonly used index to characterize obesity, but is known to be of limited accuracy in estimating adiposity [23]. In our study, *PEMT* expression in adipose was a more significant predictor of percent of fat mass compared to BMI. Polymorphisms in *PEMT* are not associated with BMI, but were associated with WHR adjusted for BMI, indicating this gene's role in body fat distribution rather than overall adiposity. These polymorphisms were also associated with transcript-level expression of *PEMT* in adipose tissue. Analysis of publicly available adipose tissue *e*QTL data also confirmed this association (Figure S4). Intriguingly, the same allele of the intronic SNP rs4646343 is associated with higher expression of PEMT in adipose and higher WHR. Thus, our study suggests an important role for *cis*-regulatory SNPs in modulating *PEMT* expression, which in turn may increase genetic susceptibility to obesity by altering phospholipid composition and inducing ER stress in adipocytes.

## Supporting Information

Figure S1 **Phosphatidylcholine (PC) and Phosphatidylethanolamine (PE) biosynthesis and remodeling.** (modified from Fu S *et al.*, 2011, Nature, 473:528–531).
(PDF)

Figure S2 **Linkage Disequilibrium among SNPs in** ***PEMT*** **gene.** Top graph also indicates association of PEMT SNPs with WHR (adjusted for BMI) in Caucasian subjects from the GIANT consortium.
(PDF)

Figure S3 **SNPs in** ***PEMT*** **gene region (±500 Kb) are associated with waist-to-hip ratio (WHR) adjusted for BMI.** Data represents a meta-analysis of 77158 Caucasian subjects from the GIANT consortium.
(PDF)

Figure S4 **Association of** ***PEMT*** **expression with local SNP genotype in MuTHER consortium-TwinUK data set.** A) Expression of *PEMT* (Illumina expression probe ILMN_1745806) in adipose is associated with local regulatory SNPs and association peaks within the intron of *PEMT*. Association of 540 SNPs within ±500 Kb of *PEMT* gene is shown (Data downloaded from Genevar database; Grundberg E et al, Nature genetics 2012; 44:1084-9). B) WHR associated intronic SNP rs4646343 is an adipose tissue specific *cis*-regulator for *PEMT* and not for any other gene within ±500 Kb. A, adipose; L, transformed lymphocyte; S, skin fibroblast.
(PDF)

Table S1 **Primer sequences for quantitative real time PCR (qRT-PCR) assays.**
(PDF)

Table S2 **Primer sequences for genotyping by pyrosequencing.**
(PDF)
