# Correction: Phospholipid Biosynthesis Genes and Susceptibility to Obesity: Analysis of Expression and Polymorphisms

**DOI:** 10.1371/annotation/7b3edc45-39c7-416b-a7dc-124f4846303d

**Published:** 2013-12-10

**Authors:** Neeraj K. Sharma, Kurt A. Langberg, Ashis K. Mondal, Swapan K. Das

In Table 3, the letter R was mistakenly removed from the beginning of each SNP during the production process. The location of rs7946 was also noted incorrectly as 7409560; it should be 17409560. Please see the corrected Table 3 here: http://plosone.org/corrections/pone.0065303.t003.cn.doc

