# Supplementary material for: Phospholipid Biosynthesis Genes and Susceptibility to Obesity: Analysis of Expression and Polymorphisms
Source: PLoS One. 2013 May 28;8(5):e65303. doi: 10.1371/journal.pone.0065303 (PMC3665552; doi:10.1371/journal.pone.0065303)

Figure S1: Phosphatidylcholine (PC) and Phosphatidylethanolamine (PE) biosynthesis and remodeling (modified from Fu S *et al.*, 2011, Nature, 473:528-531).

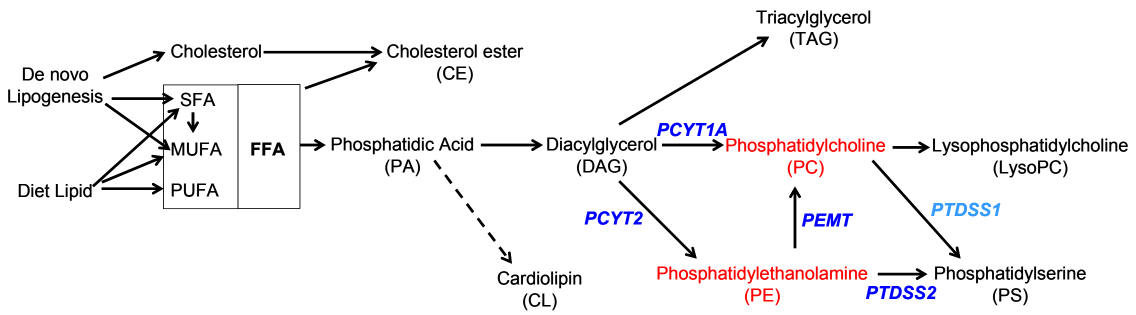

Supplement: Figure S1 — Phosphatidylcholine (PC) and Phosphatidylethanolamine (PE) biosynthesis and remodeling. (modified from Fu S et al., 2011, Nature, 473:528–531). (PDF) [file pone.0065303.s001.pdf]
