# Supplementary material for: Phospholipid Biosynthesis Genes and Susceptibility to Obesity: Analysis of Expression and Polymorphisms
Source: PLoS One. 2013 May 28;8(5):e65303. doi: 10.1371/journal.pone.0065303 (PMC3665552; doi:10.1371/journal.pone.0065303)

Figure S2: Linkage Disequilibrium among SNPs in *PEMT* gene. Top graph also indicates association of PEMT SNPs with WHR (adjusted for BMI) in Caucasian subjects from the GIANT consortium.

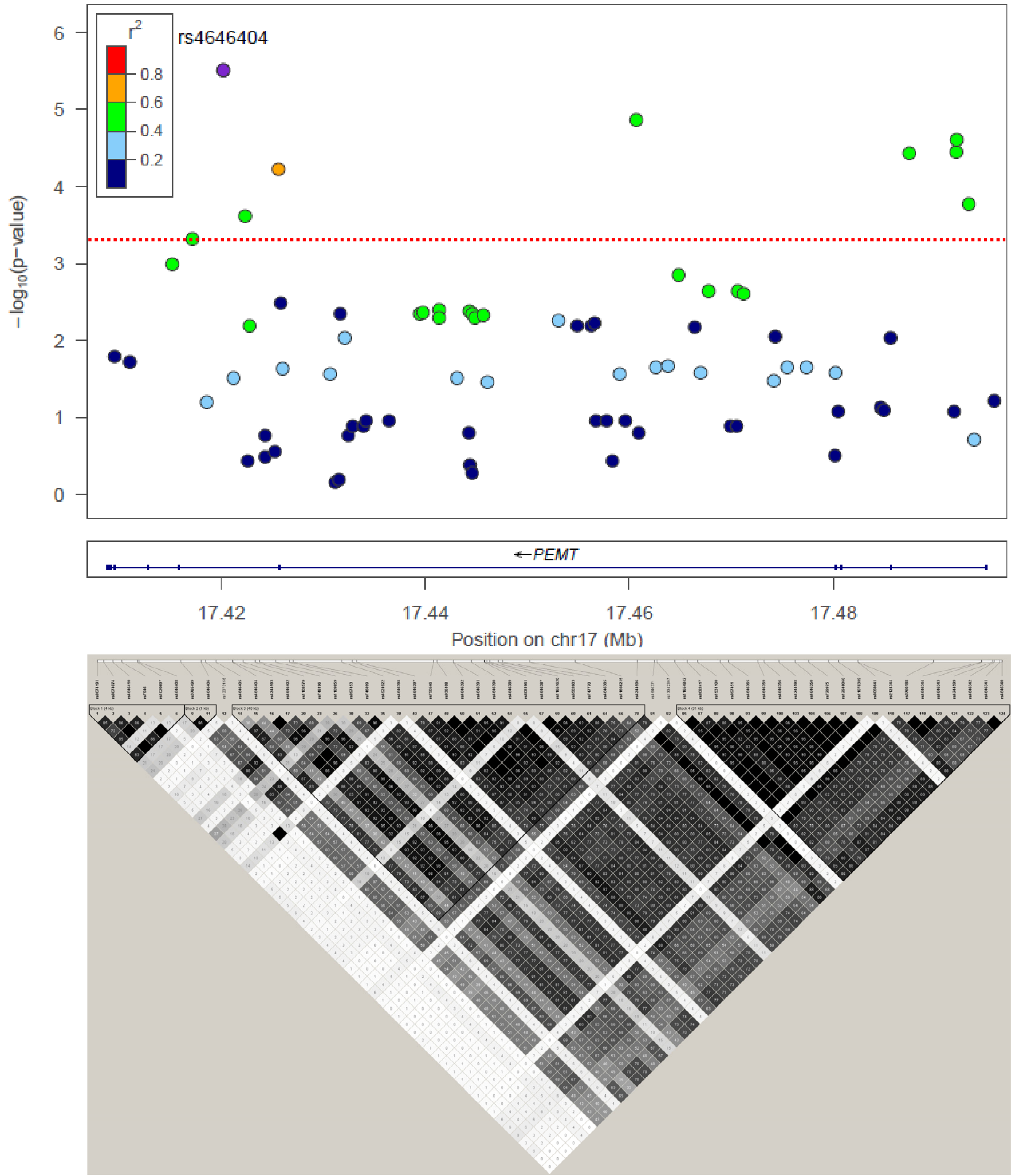

Supplement: Figure S2 — Linkage Disequilibrium among SNPs in PEMT gene. Top graph also indicates association of PEMT SNPs with WHR (adjusted for BMI) in Caucasian subjects from the GIANT consortium. (PDF) [file pone.0065303.s002.pdf]
