# Supplementary material for: Phospholipid Biosynthesis Genes and Susceptibility to Obesity: Analysis of Expression and Polymorphisms
Source: PLoS One. 2013 May 28;8(5):e65303. doi: 10.1371/journal.pone.0065303 (PMC3665552; doi:10.1371/journal.pone.0065303)

Figure S3: SNPs in *PEMT* gene region (±500 Kb) are associated with waist-to-hip ratio (WHR) adjusted for BMI. Data represents a meta-analysis of 77158 Caucasian subjects from the GIANT consortium.

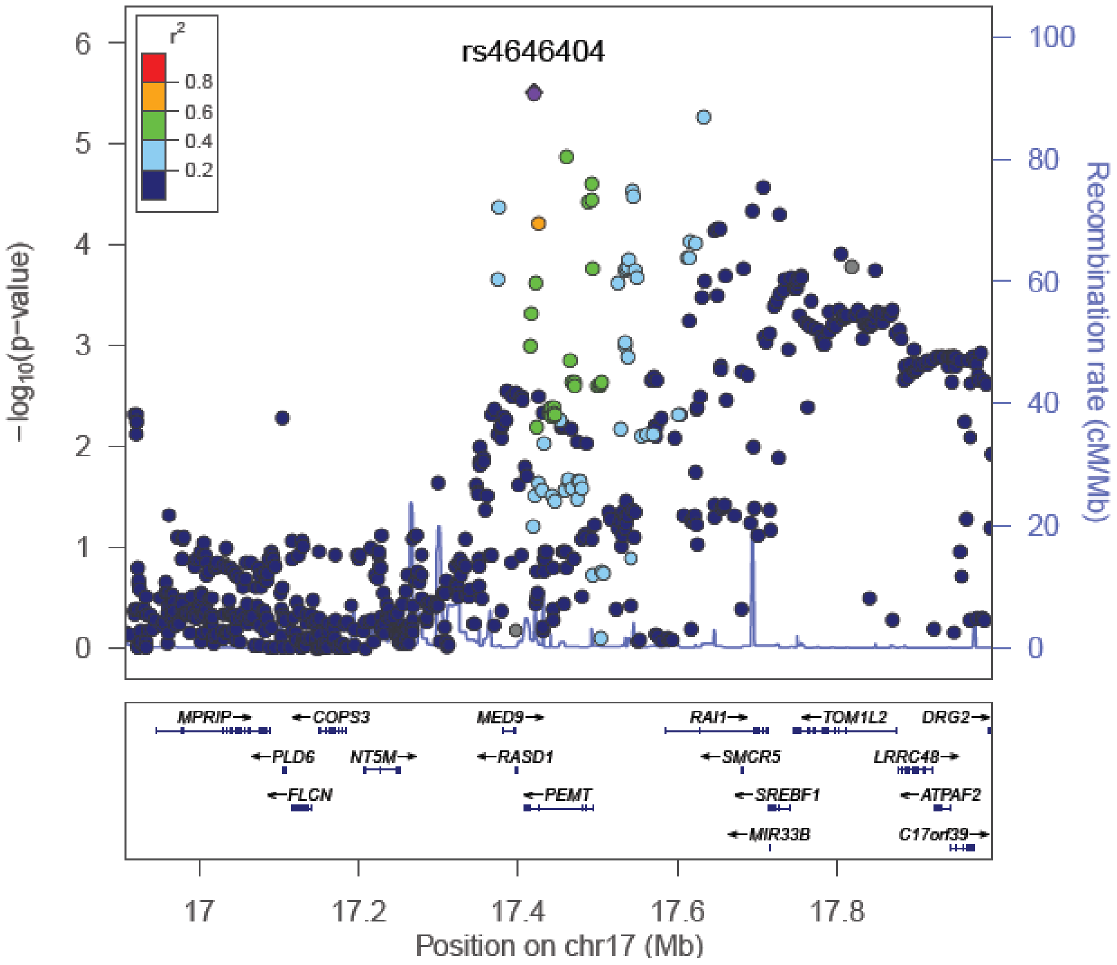

Supplement: Figure S3 — SNPs in PEMT gene region (±500 Kb) are associated with waist-to-hip ratio (WHR) adjusted for BMI. Data represents a meta-analysis of 77158 Caucasian subjects from the GIANT consortium. (PDF) [file pone.0065303.s003.pdf]
