# Supplementary material for: Phospholipid Biosynthesis Genes and Susceptibility to Obesity: Analysis of Expression and Polymorphisms
Source: PLoS One. 2013 May 28;8(5):e65303. doi: 10.1371/journal.pone.0065303 (PMC3665552; doi:10.1371/journal.pone.0065303)

Figure S4: Association of *PEMT* expression with local SNP genotype in MuTHER consortium-TwinUK data set. A) Expression of *PEMT* (Illumina expression probe ILMN_1745806) in adipose is associated with local regulatory SNPs and association peaks within the intron of *PEMT*. Association of 540 SNPs within ±500 Kb of *PEMT* gene is shown (Data downloaded from Genevar database; Grundberg E et al, Nature genetics 2012; 44:1084-9). B) WHR associated intronic SNP rs4646343 is an adipose tissue specific *cis*-regulator for *PEMT* and not for any other gene within ±500 Kb. A, adipose; L, transformed lymphocyte; S, skin fibroblast.

A:

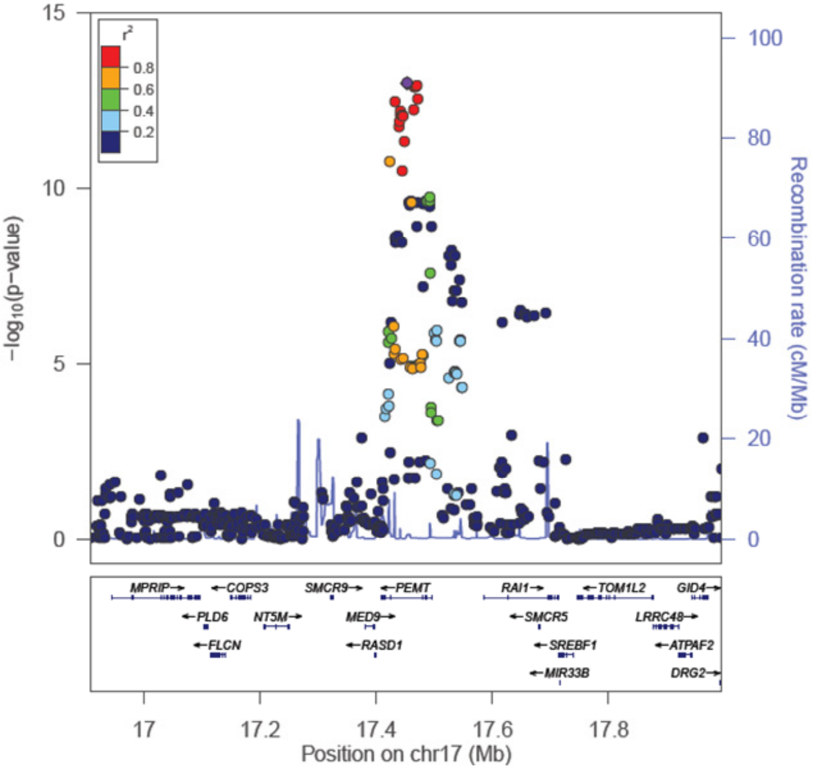

B:

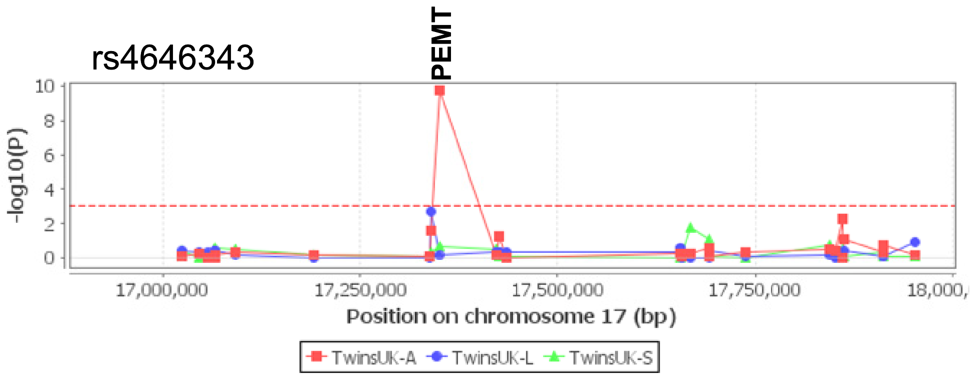

Supplement: Figure S4 — Association of PEMT expression with local SNP genotype in MuTHER consortium-TwinUK data set. A) Expression of PEMT (Illumina expression probe ILMN_1745806) in adipose is associated with local regulatory SNPs and association peaks within the intron of PEMT. Association of 540 SNPs within ±500 Kb of PEMT gene is shown (Data downloaded from Genevar database; Grundberg E et al, Nature genetics 2012; 44:1084-9). B) WHR associated intronic SNP rs4646343 is an adipose tissue specific cis-regulator for PEMT and not for any other gene within ±500 Kb. A, adipose; L, transformed lymphocyte; S, skin fibroblast. (PDF) [file pone.0065303.s004.pdf]
