# Supplementary material for: Phospholipid Biosynthesis Genes and Susceptibility to Obesity: Analysis of Expression and Polymorphisms
Source: PLoS One. 2013 May 28;8(5):e65303. doi: 10.1371/journal.pone.0065303 (PMC3665552; doi:10.1371/journal.pone.0065303)
Supplement: Table S1 — Primer sequences for quantitative real time PCR (qRT-PCR) assays. (PDF) [file pone.0065303.s005.pdf]

Table S1: Primer sequences for quantitative real time PCR (qRT-PCR) assays

Gene symbol	Entraz gene Id		Primer sequence 5'>3'
PCYT1A	5130	F	CTCCACATCAGACATCATCACC
		R	TTACTTTGTCAACCCTCTCCTG
PCYT2	5833	F	CTCACCACAGACCTCATCGT
		R	GCTCCTTGGCTTCCTTCTTC
PEMT	10400	F	TCACCATCACCTTCAATCCG
		R	GCAGGAAGTTCAGGAGCAG
PTDSS2	81490	F	TGTACGCTTGGCTATGTGAC
		R	TGGATGAGGTCTGGAAAATGG

F, Forward primer; R, Reverse primer
