# Supplementary material for: Phospholipid Biosynthesis Genes and Susceptibility to Obesity: Analysis of Expression and Polymorphisms
Source: PLoS One. 2013 May 28;8(5):e65303. doi: 10.1371/journal.pone.0065303 (PMC3665552; doi:10.1371/journal.pone.0065303)
Supplement: Table S2 — Primer sequences for genotyping by pyrosequencing. (PDF) [file pone.0065303.s006.pdf]

Table S2: Primer sequences for genotyping by pyrosequencing.

Gene name	SNP		Primers (5'>3')
PEMT	rs7946	F-M13	CACGACGTTGTAAAACGACCAGCCCCTACTCACTCTTCGT
		R	TGACCTGCCTCTGTCCTTTC
		Seq	GGCCCTCACCTACATA
PEMT	rs4646343	F	CCGGTGGCAGATGGATGAGAG
		R-M13	CACGACGTTGTAAAACGACGGTGATTCGCTATGGAAAAGG
		Seq	TCTTGCTGTCCACAGG
PEMT	rs897453	F-M13	CACGACGTTGTAAAACGACGGAACACAAGACCCGCAAG
		R	GCGTGGTGAGGGATGAGGT
		Seq	AGGAGCAGGATGGTG
PEMT	rs4646404	F-M13	CACGACGTTGTAAAACGACGCTTTTGGTCTCTCTGGCTTC
		R	CGCCCCACCCCTTACTCTCC
		Seq	GGACCCCGCCCAGTG

F, Forward primer; R, Reverse primer; M13, primer with 19bp 5'-M13 universal tail; Seq, Pyrosequencing primer.
